# A Population-Level Analysis of the Protective Effects of Androgen Deprivation Therapy Against COVID-19 Disease Incidence and Severity

**DOI:** 10.3389/fmed.2022.774773

**Published:** 2022-05-04

**Authors:** Kyung Min Lee, Kent Heberer, Anthony Gao, Daniel J. Becker, Stacy Loeb, Danil V. Makarov, Barbara Gulanski, Scott L. DuVall, Mihaela Aslan, Jennifer Lee, Mei-Chiung Shih, Julie A. Lynch, Richard L. Hauger, Matthew Rettig

**Affiliations:** ^1^VA Informatics and Computing Infrastructure, VA Salt Lake City Health Care System, Salt Lake City, UT, United States; ^2^Division of Epidemiology, Department of Internal Medicine, University of Utah School of Medicine, Salt Lake City, UT, United States; ^3^VA Palo Alto Healthcare System, Palo Alto, CA, United States; ^4^Department of Medicine, Stanford University School of Medicine, Palo Alto, CA, United States; ^5^VA New York Harbor Healthcare System, New York, NY, United States; ^6^Department of Medicine, Perlmutter Cancer Center, New York University Langone Health, New York, NY, United States; ^7^Department of Urology, Perlmutter Cancer Center, New York University Langone Health, New York, NY, United States; ^8^Cooperative Studies Program Clinical Epidemiology Research Center (CSP CERC), VA Connecticut Healthcare System, West Haven, CT, United States; ^9^Department of Internal Medicine, Yale School of Medicine, Yale University, New Haven, CT, United States; ^10^Department of Biomedical Data Science, Stanford University School of Medicine, Palo Alto, CA, United States; ^11^Center of Excellence for Stress and Mental Health (CESAMH), VA San Diego Healthcare System, San Diego, CA, United States; ^12^Department of Psychiatry, Center for Behavior Genetics of Aging, School of Medicine, University of California, San Diego, La Jolla, CA, United States; ^13^VA Greater Los Angeles Healthcare System, Los Angeles, CA, United States; ^14^School of Medicine, University of California, Los Angeles, Los Angeles, CA, United States

**Keywords:** androgen deprivation therapy, COVID-19 incidence, COVID-19 severity, prostate cancer, Veterans Health Administration

## Abstract

**Background:**

The incidence and severity of coronavirus disease 19 (COVID-19) is substantially higher in men. Sex hormones may be a potential mechanism for differences in COVID-19 outcome in men and women. We hypothesized that men treated with androgen deprivation therapy (ADT) have lower incidence and severity of COVID-19.

**Methods:**

We conducted an observational study of male Veterans treated in the Veterans Health Administration from February 15th to July 15th, 2020. We developed a propensity score model to predict the likelihood to undergo Severe Acute Respiratory Syndrome Coronavirus 2 (SARS-CoV-2) testing. We performed multivariable logistic regression modeling adjusted with inverse probability weighting to examine the relationship between ADT and COVID-19 incidence. We conducted logistic regression analysis among COVID-19 patients to test the association between ADT and COVID-19 severity.

**Results:**

We identified a large cohort of 246,087 VA male patients who had been tested for SARS-CoV-2, of whom 3,057 men were exposed to ADT, and 36,096 men with cancer without ADT. Of these, 295 ADT patients and 2,427 cancer patients not on ADT had severe COVID-19 illness. In the primary, propensity-weighted comparison of ADT patients to cancer patients not on ADT, ADT was associated with decreased likelihood of testing positive for SARS-CoV-2 (adjusted OR, 0.88 [95% CI, 0.81–0.95]; *p* = 0.001). Furthermore, ADT was associated with fewer severe COVID-19 outcomes (OR 0.72 [95% CI 0.53–0.96]; *p* = 0.03).

**Conclusion:**

ADT is associated with reduced incidence and severity of COVID-19 amongst male Veterans. Testosterone and androgen receptor signaling may confer increased risk for SARS-CoV-2 infection and contribute to severe COVID-19 pathophysiology in men.

## Introduction

Severe acute respiratory syndrome coronavirus 2 (SARS-CoV-2) infection and coronavirus disease 2019 (COVID-19) have rapidly disseminated across the globe, accelerated with the delta variant surge, and caused more than 4.6 million deaths (1). The incidence and severity of COVID-19 have been related to multiple factors, including age, comorbid conditions, immunosuppression, smoking, race, and sex (2–4). Whereas men and women manifest a similar incidence of COVID-19, male sex is a risk factor for more severe illness. The severity of COVID-19 as indicated by admission to an intensive care unit (ICU), requirement for mechanical ventilation, or mortality is approximately twofold higher in men (5, 6). This sex disparity has been observed in Asia, North America, Europe, and across individual countries on each of the continents.

Explanations for sex differences in outcome include difference in health behaviors (e.g., smoking history), incidence of comorbidities such as lung and cardiovascular disease, genetics, and biology, including variations in sex hormones. Specifically, androgens including testosterone and dihydrotestosterone may worsen COVID-19 severity through two non-mutually exclusive physiologic effects. First, androgens are known to suppress both innate and adaptive immunity, which may increase susceptibility to the SARS-CoV-2 virus and disease severity (7, 8). Second, androgens induce the expression of the two plasma membrane proteins, transmembrane protease serine 2 (TMPRSS2) *via* an androgen response element in the promoter and angiotensin converting enzyme 2 (ACE2), which are required for entry of SARS-CoV-2 into epithelial cells (9–12). Co-expression of the androgen receptor (AR), TMPRSS2, and ACE2 has been observed in human pulmonary epithelial cells, alveolar type 2 pneumocytes, and nasal mucosal cells which are critical sites for SARS-CoV-2 infection and disease severity (12, 13). Androgen-driven overexpression of these viral co-receptors may augment cellular entry and replication of SARS-CoV-2, thereby mediating increased infection rate and COVID-19 severity in men. Furthermore, men who are carriers of TMPRSS2 gene mutations, which potentiate androgen-induced cellular expression of TMPRSS2, may be especially at risk for COVID-19 morbidity and mortality (14, 15). TMPRSS2 gene variants has also been found to confer a twofold increase in H1N1 influenza severity supporting the hypothesis that androgen regulation of TMPRSS2 may be a critical host factor for respiratory disease (16).

These observations suggest that suppression of androgen levels may have a salutary impact on COVID-19 illness. Two retrospective studies that evaluated the impact of androgen deprivation therapy (ADT) on COVID-19 illness yielded conflicting results. A study of 4,532 men in the Veneto region of Italy reported a reduced incidence of COVID-19 among patients with prostate cancer on ADT, whereas a study of patients in the United States (U.S.) failed to detect a protective effect of ADT on SARS-CoV-2 infection, but did not have sufficient statistical power to assess the effect of ADT on COVID-19 severity (17, 18). There have also been three additional investigations but with small sample sizes with one report finding that hospitalization with severe COVID-19 was significantly less in prostate cancer patients treated with ADT compared to those without ADT while two other reports observed no differences in severe COVID-19 between ADT and non-ADT groups (19–21). Our objective was to perform a nationwide, population-based study of the relationship of ADT to the incidence and outcomes of COVID-19. Using data from the U.S. Veterans Health Administration (VHA), we report the largest study to date evaluating the hypothesis that ADT is associated with a reduced incidence and severity of COVID-19.

## Materials and Methods

### Data Sources

We used the Corporate Data Warehouse (CDW) of the VHA, a national data repository that provides access to the electronic health records of all individuals who received care in the VHA. In addition, we drew from the VA COVID-19 Shared Data Resource (CSDR), a newly developed data domain that consists of a wide range of information related to COVID-19 available for all patients who received a COVID-19 laboratory test within VHA or whose positive test result outside VHA was recorded in VHA clinical notes (22). This study was approved by the VA Central Institutional Review Board.

### Study Sample

The study sample consisted of all male Veterans who were alive as of February 15, 2020. The earliest testing date reported in the CSDR is February 16, 2020. Therefore, we considered anyone alive as of February 15, 2020 as having been eligible to be tested for SARS-CoV-2. From this base sample, we assembled two sub-samples to examine incidence of COVID-19 and severity of COVID-19. For incidence analysis, we derived a sample consisting of patients who underwent SARS-CoV-2 testing matched to patients who did not undergo SARS-CoV-2 testing on age, race, and VHA facility. Separately, we used an unmatched sample of patients who tested positive for SARS-CoV-2 to investigate ADT use and COVID-19 severity. The index date for the incidence analysis and the severity analysis was February 15, 2020 and the date of first positive SARS-CoV-2 test result, respectively.

### Exposure

Exposure to ADT was defined as having any prescription for a luteinizing hormone releasing hormone analog (LHRH) (buserelin, degarelix, goserelin, historelin, leuprolide, or triptorelin) or an antiandrogen (abiraterone, apalutamide, bicalutamide, cyproterone, darolutamide, enzalutamide, flutamide, ketoconazole, or nilutamide) in the 6 months prior to the index date. A small number of patients were prescribed only antiandrogens. These patients were presumed to be receiving their LHRH analogs outside the VHA because it is highly unlikely to be prescribed antiandrogens as a monotherapy.

### Endpoints

We investigated the relationship between ADT use and COVID-19 incidence and severity. To assess incidence, we used a binary variable indicating any positive reverse transcriptase polymerase chain reaction (RT-PCR) SARS-CoV-2 test result through July 15, 2020. To measure severity, we constructed a binary variable indicating whether a patient was admitted to the intensive care unit (ICU), placed on mechanical ventilation, or dead in the 60 days following a positive test up to July 15, 2020. Death was attributed to COVID-19 if it occurred within 60 days of a positive RT-PCR test for SARS-CoV-2. The primary comparison for both incidence and severity was patients with ADT exposure (ADT patients) vs. patients with non-prostate cancer who had no ADT exposure (non-ADT, other cancer patients). We selected this as the primary comparison because the incidence and severity of COVID-19 is adversely affected by a cancer diagnosis and virtually all ADT patients have high risk, recurrent, locally advanced, or metastatic prostate cancer. Secondary comparisons included all patients exposed to ADT vs. all who were not, and patients with prostate cancer with vs. without ADT exposure.

### Covariates

Age, race, marital status, body mass index, smoking status, the Charlson Comorbidity Index categories (23) (excluding localized and metastatic solid tumors) in the prior 2 years, use of selected antihypertensives—angiotensin-converting enzyme inhibitors (ACEs), angiotensin II receptor blockers (ARBs), and spironolactone—in the prior 6 months, VHA utilization in the prior year as measured by total numbers of visits and hospital days, and the number of days to testing or first SARS-CoV-2 positivity were assessed at the index date. Lastly, the VHA facility of COVID-19 testing was included as a fixed effect in the COVID-19 incidence models and as a random effect in the COVID-19 severity models.

### Statistical Analysis

Multivariate logistic regression models were used to determine the association of ADT exposure with SARS-CoV-2 positivity, weighted by the inverse of the predicted probabilities of being tested. Due to the limited availability of SARS-CoV-2 tests, receipt of test was prioritized based on a wide range of patient characteristics including demographics, comorbid conditions, and severity of symptoms. Such targeted testing results in a highly selected group of patients who are tested for SARS-CoV-2 (24). To adjust for unequal probabilities of being tested, we used the inverse probability weighting method, where the weight is based on the predicted probabilities (propensity score) of being tested, estimated by a logistic regression model using ADT exposure, selected patient covariates, and VHA facility (25, 26). For this model, we implemented a nested case-control design with incidence density sampling to match each tested patient (case) to five patients who were eligible to be tested (controls) at the time of the case’s testing on age, race, and VHA facility (27).

Generalized linear mixed models were used to determine associations between ADT exposure and severity of COVID-19 while controlling for patients’ demographic and clinical covariates, and the number of days from February 15 to the date of first SARS-CoV-2 positivity (as fixed effects) and VHA facility (as a random effect). All male Veterans in the study sample with a positive COVID-19 test were included in this analysis. We used SAS 9.2 (Cary, NC) for data preparation and all statistical analyses. Two-tailed hypothesis testing was performed using the significance level of 5%.

## Results

### Patient Characteristics

We identified 246,087 male patients tested for SARS-CoV-2 through July 15, 2020 (Table 1). Among the 246,087 tested patients, 3,057 patients were prescribed ADT and 243,030 were not. Compared to non-ADT patients, ADT patients were more likely to be Black (ADT 37%, no ADT 24%), older (mean age 73.9 vs. 62.9; *p* < 0.001), and had more comorbidities (Charlson Comorbidity Index ≥ 5 in 72% of ADT patients and 28% of non-ADT patients).

**TABLE 1:** Baseline characteristics of patients tested for SARS-CoV-2.

Characteristic	ADT*^a^ N* = 3,057		No ADT *N* = 243,030		*P*-value*^b^*	No ADT (other cancer) *N* = 36,096		*P*-value*^b^*
Age (SD)	73.9	(9)	62.9	(15.0)	<0.001	70.2	(10)	<0.001
Race (%)					<0.001			<0.001
White, non-Hispanic	1,614	(53)	150,987	(62)		25,726	(71)	
White, Hispanic	146	(5)	14,702	(6)		1,619	(4)	
Black	1,128	(37)	57,286	(24)		6,606	(18)	
Other	35	(1)	5,174	(2)		508	(1)	
Unknown	134	(4)	14,881	(6)		1,637	(5)	
Marital status (%)					<0.001			<0.001
Married	1,479	(48)	111,052	(46)		17,229	(48)	
Single	336	(11)	40,741	(17)		4,334	(12)	
Separated or divorced	943	(31)	76,979	(32)		11,742	(33)	
Widowed	290	(9)	12,461	(5)		2,658	(7)	
Unknown	<11	(0)	1,797	(1)		133	(0)	
BMI (SD)	29.0	(6)	29.8	(6.5)	<0.001	28.7	(6)	0.062
Smoking status (%)					<0.001			<0.001
Current	845	(28)	79,733	(33)		11,459	(32)	
Former	1,896	(62)	129,073	(53)		21,800	(60)	
Never	316	(10)	34,224	(14)		2,837	(8)	
**Charlson comorbidity index**								
*Score*					<0.001			<0.001
0	22	(1)	68,330	(28)		2,082	(6)	
1	< 11	(0)	33,924	(14)		1,847	(5)	
2	298	(10)	25,838	(11)		4,065	(11)	
3	295	(10)	26,139	(11)		4,412	(12)	
4	239	(8)	20,150	(8)		3,709	(10)	
≥ 5	2,199	(72)	68,649	(28)		19,981	(55)	
***Categories* (%)**								
Cerebrovascular disease	473	(15)	29,599	(12)	<0.001	6,390	(18)	0.002
Congestive heart failure	672	(22)	38,749	(16)	<0.001	8,410	(23)	0.098
Chronic pulmonary disease	949	(31)	66,251	(27)	<0.001	14,534	(40)	<0.001
Dementia	507	(17)	24,562	(10)	<0.001	5,109	(14)	0.000
Diabetes without chronic complication	1,310	(43)	84,246	(35)	<0.001	15,159	(42)	0.357
Diabetes with chronic complication	918	(30)	57,977	(24)	<0.001	11,202	(31)	0.249
Hemiplegia or paraplegia	76	(2)	4,931	(2)	0.075	967	(3)	0.525
HIV/AIDS	34	(1)	2,995	(1)	0.549	555	(2)	0.064
Mild liver disease	338	(11)	29,782	(12)	0.045	6,208	(17)	<0.001
Severe liver disease	166	(5)	14,830	(6)	0.123	3,556	(10)	<0.001
Myocardial Infarction	256	(8)	15,120	(6)	<0.001	3,367	(9)	0.081
Peptic ulcer disease	60	(2)	4,473	(2)	0.618	1,128	(3)	<0.001
Peripheral vascular disease	632	(21)	36,990	(15)	<0.001	8,624	(24)	<0.001
Renal disease	1,107	(36)	53,493	(22)	<0.001	12,538	(35)	0.100
Rheumatic disease	64	(2)	4,897	(2)	0.759	988	(3)	0.035
**Medications (%)**								
ACE	873	(29)	57,952	(24)	<0.001	9,599	(27)	0.019
ARB	369	(12)	24,896	(10)	<0.001	4,311	(12)	0.835
Spironolactone	134	(4)	8,269	(3)	0.003	1,682	(5)	0.485
**VHA utilization in the prior year**								
Outpatient visits (SD)	45.8	(35.0)	32.6	(36.2)	<0.001	43.7	(38.5)	<0.001
Inpatient days (SD)	7.1	(26.7)	6.8	(28.8)	<0.001	8.9	(30.7)	0.008
Days to SARS-CoV-2 testing from 2/15/2020 (SD)	103.4	(31.1)	104.3	(33.1)	0.011	102.9	(32.4)	0.803

*ADT, Androgen Deprivation Therapy; SD, Standard Deviation; BMI, Body Mass Index; ACE, Angiotensin-Converting Enzyme inhibitors; ARB, Angiotensin II Receptor Blockers; VHA, Veterans Health Administration; SARS-CoV-2, Severe acute respiratory syndrome coronavirus 2. Regulations prohibit displaying cell sizes of less than 11.*

*^a^Defined as having any prescriptions for LHRH analogs or antiandrogens in the 6 months prior to 2/15/2020.*

*^b^Calculated using the Wilcoxon rank-sum test (continuous variables) or chi-square test (categorical variables).*

### Severe Acute Respiratory Syndrome Coronavirus 2 Positivity

**FIGURE 1:**
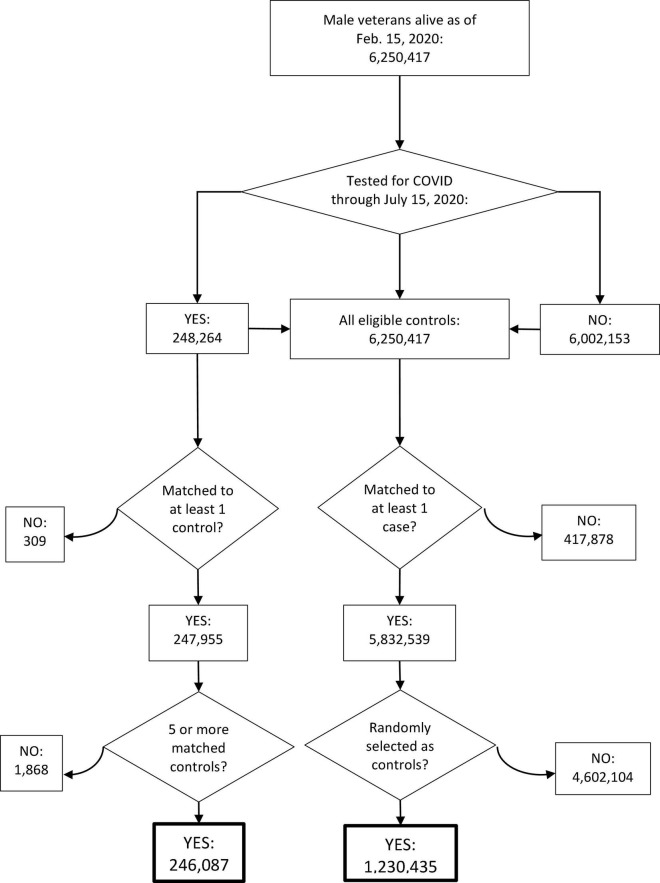
Consort diagram for the selection of patients for the model for the propensity to be tested for SARS-CoV-2. Curved arrows indicate excluded patients.

The strategy for selecting patients to be included in the SARS-CoV-2 testing propensity score model is shown in Figure 1, and the baseline characteristics of all SARS-CoV-2-tested patients (cases) and the matched controls are shown in Supplementary Table 1. ADT patients were more likely to be tested for SARS-CoV-2 (adjusted odds ratio (OR), 1.59 [95% confidence interval (CI), 1.52–1.68]; Supplementary Table 2). In the primary, propensity-weighted comparison of ADT vs. cancer patients not on ADT, ADT was associated with decreased likelihood of testing positive for SARS-CoV-2 after adjusting for the covariates listed in Table 1 (OR, 0.88 [95% CI, 0.81–0.95]; *p* = 0.001; Table 2). Similar results were observed in secondary comparisons of ADT patients vs. all non-ADT patients (OR, 0.75 [95% CI, 0.70–0.81]; *p* < 0.001; Table 2) and patients with prostate cancer on ADT vs. patients with prostate cancer not on ADT (OR, 0.85 [95% CI, 0.77–0.94]; *p* = 0.002; Supplementary Table 3).

**TABLE 2:** Association between ADT use and SARS-CoV-2 positivity.

Comparison	ADT group	SARS-CoV-2 positive (%)	No. tested	Reference group	SARS-CoV-2 positive (%)	No. tested	OR (95% CI)	*P*-value
Primary	ADT	233 (8)	3,057	Other cancer (No ADT)	2,385 (7)	36,096	0.88 (0.81,0.95)	0.001
Secondary	ADT	233 (8)	3,057	No ADT	23,340 (10)	243,030	0.75 (0.70,0.81)	<0.001

*ADT, Androgen Deprivation Therapy; SARS-CoV-2, Severe acute respiratory syndrome coronavirus 2.*

### Coronavirus Disease 19 Severity

Our primary comparison of COVID-19 severity included 295 ADT patients with COVID-19 (including 189 with prostate cancer only and 97 with prostate cancer and a second cancer diagnosis), and 2,427 non-ADT, other cancer patients with COVID-19. Significant differences between the two groups included age, race, smoking status, and comorbidities (Table 3). Severe COVID-19 outcomes were observed in 76/295 (25%) of the ADT group and 727/2427 (30%) of the non-ADT group (Table 4). After controlling for clinical and demographic factors, ADT was associated with fewer severe COVID-19 outcomes (OR 0.72 [95% CI 0.53–0.96]; *p* = 0.03; Table 4). Comparisons between ADT patients vs. all patients not on ADT and prostate cancer patients on ADT vs. prostate cancer patients not on ADT did not demonstrate a statistically significant protective effect of ADT (Table 4 and Supplementary Table 3).

**TABLE 3:** Baseline characteristics at first positive SARS-CoV-2 test.

Characteristic	ADT^a^ *N* = 295		No ADT *N* = 25,006		*P*-value^b^	No ADT (Other cancer) *N* = 2,427		*P*-value^b^
Age (SD)	74.2	(10.2)	62.1	(16.4)	<0.001	71.0	(11.8)	<0.001
Race (%)					<0.001			<0.001
White, non-Hispanic	111	(38)	11,619	(46)		1,386	(57)	
White, Hispanic	13	(4)	2,406	(10)		150	(6)	
Black	152	(52)	8,580	(34)		752	(31)	
Other	<11	(0)	659	(3)		33	(1)	
Unknown	18	(6)	1,742	(7)		106	(4)	
Marital status (%)					0.007			0.484
Married	141	(48)	11,686	(47)		1,134	(47)	
Single	43	(15)	4,460	(18)		316	(13)	
Separated or divorced	84	(28)	7,321	(29)		761	(31)	
Widowed	27	(9)	1,278	(5)		200	(8)	
Unknown	<11	(0)	261	(1)		16	(1)	
BMI (SD)	28.8	(6.2)	30.4	(6.5)	<0.001	28.7	(6.5)	0.617
Smoking status (%)					0.003			0.326
Current	71	(24)	5,927	(24)		650	(27)	
Former	191	(65)	14,386	(58)		1,561	(64)	
Never	33	(11)	4,693	(19)		216	(9)	
Charlson Comorbidity index								
*Score*					<0.001			<0.001
0	<11	(0)	8,004	(32)		175	(7)	
1	<11	(0)	3,868	(15)		147	(6)	
2	33	(11)	2,597	(10)		277	(11)	
3	25	(8)	2,847	(11)		301	(12)	
4	27	(9)	1,876	(8)		254	(10)	
≥ 5	208	(71)	5,814	(23)		1,273	(52)	
***Categories* (%)**								
Cerebrovascular disease	62	(21)	2,815	(11)	<0.001	447	(18)	0.280
Congestive heart failure	77	(26)	3,314	(13)	<0.001	576	(24)	0.368
Chronic pulmonary disease	91	(31)	5,160	(21)	<0.001	843	(35)	0.184
Dementia	57	(19)	3,212	(13)	0.001	539	(22)	0.258
Diabetes without chronic complication	133	(45)	9,084	(36)	0.002	1,085	(45)	0.902
Diabetes with chronic complication	92	(31)	5,890	(24)	0.002	804	(33)	0.503
Hemiplegia or paraplegia	<11	(3)	434	(2)	0.087	75	(3)	0.971
HIV/AIDS	<11	(1)	333	(1)	0.800*^c^*	43	(2)	0.605
Mild liver disease	33	(11)	2,369	(9)	0.318	370	(15)	0.064
Severe liver disease	22	(7)	1,092	(4)	0.010	201	(8)	0.626
Myocardial Infarction	24	(8)	1,088	(4)	0.002	199	(8)	0.970
Peptic ulcer disease	<11	(3)	295	(1)	0.026*^c^*	63	(3)	0.906
Peripheral vascular disease	62	(21)	3,040	(12)	<0.001	527	(22)	0.784
Renal disease	110	(37)	4,987	(20)	<0.001	844	(35)	0.393
Rheumatic disease	<11	(1)	375	(1)	0.335*^c^*	49	(2)	0.109
**Medications (%)**								
ACE	90	(27)	5,134	(21)	0.005	529	(22)	0.038
ARB	39	(13)	2,489	(10)	0.063	296	(12)	0.613
Spironolactone	<11	(3)	618	(2)	0.525	89	(4)	0.592
**VHA utilization in the prior year**								
Outpatient visits (SD)	46.2	(42.0)	26.9	(34.7)	<0.001	42.1	(43.4)	<0.001
Inpatient days (SD)	8.2	(29.9)	7.0	(35.7)	<0.001	14.2	(50.9)	0.668
Days to SARS-CoV-2 positivity from 2/15/2020 (SD)	96.2	(38.9)	101.0	(37.7)	0.022	95.6	(37.2)	0.972

*ADT, Androgen Deprivation Therapy; SD, Standard Deviation; BMI, Body Mass Index; CCI, Charlson Comorbidity Index; ACE, Angiotensin-converting enzyme inhibitors; ARB, Angiotensin II Receptor Blockers; SARS-CoV-2, Severe acute respiratory syndrome coronavirus 2. VHA Data User Agreement prohibits displaying cell sizes of less than 11.*

*^a^Defined as having any prescriptions for luteinizing hormone releasing hormone analogs or antiandrogens in the 6 months prior to the date of first positive SARS-CoV-2 test.*

*^b^Calculated using the Wilcoxon rank-sum test (continuous variables) or chi-square test (categorical variables).*

*^c^Calculated using the two-sided Fisher’s exact test.*

**TABLE 4:** Association between ADT use and COVID-19 severity.

Comparison	ADT group	Severe^a^ (%)	No. positive	Reference group	Severe^a^ (%)	No. positive	OR (95% CI)	*P*-value
Primary	ADT	76 (26)	295	Other cancer (No ADT)	727 (30)	2,427	0.72 (0.53,0.96)	0.025
Secondary	ADT	76 (26)	295	No ADT	4,363 (17)	25,006	0.88 (0.66,1.17)	0.372

*ADT, Androgen Deprivation Therapy; COVID-19, Coronavirus Disease 2019.*

*^a^Severe is defined as ICU admission, mechanical ventilation, or death in the 60 days following SARS-CoV-2 positivity.*

## Discussion

In our analysis of male U.S. Veterans tested for SARS-CoV-2, ADT use was associated with a reduced incidence and severity of COVID-19 compared to Veterans with non-prostate cancers who were not on ADT. The reduction in the incidence of COVID-19 was modest (OR 0.88) after correcting for baseline characteristics (e.g., smoking, cardiovascular disease and other comorbidities, and race) and the likelihood for undergoing SARS-CoV-2 testing. A reduced incidence of COVID-19 was also observed among ADT patients compared to all SARS-CoV-2-tested Veterans not on ADT as well as Veterans with prostate cancer not on ADT.

The effects of ADT on the incidence of COVID-19 has been reported in two studies. Montopoli et al. (17) evaluated SARS-CoV-2 positive patients in the Veneto region of Italy and reported that COVID-19 infection was significantly higher in patients with a cancer diagnosis other than prostate cancer as well as patients with prostate cancer not on ADT compared to prostate cancer patients treated with ADT (OR 4.86 [95% CI 1.88–12.56]; *p* = 0.001, and OR 4.05 [95% CI 1.55–10.59]; *p* = 0.004, respectively). These results were based on COVID-19 incidence among all patients with cancer in the Veneto region, irrespective of whether these patients had been tested for SARS-CoV-2. Whereas the results from the Italian study are consistent with the protective effect of ADT on COVID-19 incidence in the analogous populations in the Veteran population, the magnitude of the effect was far greater in the Veneto population. In contrast, Klein et al. (18), reporting on a cohort of patients from Cleveland Clinic healthcare system, observed no protective effect of ADT on incidence of COVID-19 in prostate cancer patients on ADT vs. prostate cancer patients not on ADT.

The association of ADT with the severity of COVID-19 has been reported in three studies. In the Italian study, ADT was associated with a reduced likelihood of severe disease (ICU admission and death), but this was premised on only four COVID-19 positive patients on ADT (only 1 of whom developed severe disease), 13 COVID-19 positive patients with prostate cancer not on ADT, and 89 COVID-19 positive patients with other cancers not on ADT (17). By contrast, the Ohio study did not identify a protective effect of ADT on the severity of COVID-19 outcomes, but the authors recognized that robust statistics were not feasible due to limited sample size, despite being significantly larger than the Italian cohort (18). Based on a small cohort of ADT and non-ADT patients (*n* = 22 and 36, respectively), Patel et al. (19) reported that ADT was associated with a reduced risk of hospitalization (OR 0.23 [95% CI 0.06–0.79]; *p* < 0.02) and supplemental oxygen use (OR 0.26 [95% CI 0.07–0.92]; *p* = 0.04), and a non-statistically significant trend toward a reduction in mechanical ventilation and death after correcting for age, cardiac disease, and pulmonary disease (19). Two other reports with small sample sizes did not observe a reduction in COVID-19 severity with ADT (20, 21). By comparison, our study demonstrated ADT exposure was associated with a reduction in COVID-19 severity (OR 0.72) compared to cancer patients without ADT exposure, based on a composite endpoint of ICU admission, mechanical ventilation, and death.

Strengths of this study include a multivariate regression analysis to account for differences in baseline characteristics, which was not performed in the Italian study, as well as a much larger cohort of patients in comparison to the Italian study, though still an order of magnitude smaller than the current VHA cohort (e.g., *n* = 304 and *n* = 3,057 patients on ADT in the Ohio and Veteran cohorts, respectively). Importantly, neither the Italian nor the Ohio study accounted for differences in the likelihood to be tested for SARS-CoV-2 among the respective populations, which can bias the COVID-19 incidence results, especially if ADT patients are more likely to be tested for SARS-CoV-2. Indeed, in our Veteran population, the adjusted odds ratio for SARS-CoV-2 testing was 1.59 for ADT patients, which we incorporated as a propensity score in our analyses of COVID-19 incidence. In addition, our study is less subject to ascertainment bias, since Veterans largely seek care within the VA healthcare system. Weaknesses include the retrospective nature of the analysis and uncertainty about the underlying cause for ICU admission, mechanical ventilation, and death. While we treated VHA facility as a random effect in our severity analysis, the rapid change in treatment decisions and ability for hospitals to handle capacity at different times in the pandemic may leave confounding for patients who develop severe disease.

The role of androgens in SARS-CoV-2 cell entry and replication and in the clinical course and outcome of COVID-19 is likely to be complicated. Testosterone and dihydrotestosterone (DHT) are regulators of ACE-2, the membrane receptor binding SARS-CoV-2, and TMPRSS2, which promotes cellular entry of the virus following proteolytic processing of its spike protein (9–12). Downregulation of the expression and function of ACE-2 and TMPRSS2 in response to androgen deprivation may contribute to our data showing that ADT decreases COVID-19 infection and severity.

Androgen regulation of cellular immunity, however, may be a more important mechanism mediating our observation of ADT-induced reduction in COVID-19 infection and improvement of COVID-19 outcome. In general, androgens are immunosuppressive dampening many aspects of adaptive humoral and cell-mediated immune responses to pathogens including viruses (7, 8). Testosterone and DHT bind to androgen receptors on T lymphocytes, impair T lymphocyte activation, and inhibit Th1 differentiation and interferon-gamma (IFN-g) generation (28). Early T cell activation and interferon response are critical during the initial phase of COVID-19 infection for rapid clearance of SARS-CoV-2. Women have been found to rapidly activate T lymphocytes and increase interferon levels at the onset of COVID-19 infection, which has been shown to result in a good outcome (29). In contrast, men with low T lymphocyte activation and insufficient early interferon response impairs SARS-CoV-2 clearance and results in severe COVID-19 (29). Normal function of the JAK kinase TYK2 expressed in T lymphocytes has a critical role in T cell differentiation and type I interferon signaling (30). Testosterone and DHT bind to the promoter of the Ptnpn1 and increase the expression and function of this phosphatase (31). Ptpn1 then dephosphorylates the TYK2 protein thereby impeding TYK2-mediated T helper-1 (Th1) differentiation and interferon signaling essential for innate and acquired immune responses to viruses. Recently, a TYK2 missense mutation has been reported to confer high risk for COVID-19 severity (32). Consequently, androgen suppression of TYK2 signaling in T lymphocytes may be an important determinant of COVID-19 outcome. These findings suggest that ADT may lessen COVID-19 severity by abrogating androgen immunosuppression and promoting T cell differentiation and interferon signaling. In addition to research on androgens and cellular immunity, our hypothesis is also supported by clinical studies showing that ADT improves the response to immunotherapy of prostate cancer by counteracting androgen immunosuppression.

Alternatively, the susceptibility to COVID-19 infection and severity in men may be mediated by the interaction of androgens with the renin-angiotensin-aldosterone system (RAAS). The canonical RAAS pathway includes two angiotensin converting enzymes: (a) ACE that converts angiotensin I to octapeptide angiotensin II that binds to the Gq-coupled AT1a receptor; (b) ACE2, encoded by a gene located on the X chromosome at Xp22, that cleaves a single hydrophobic amino acid, Phe, from the C-terminus of angiotensin II to form angiotensin-(1–7), which binds to the Mas receptor, another G protein-coupled receptor in the RAAS system (33, 34). The actions of RAAS are homeostatically controlled by the AT1a receptor being counterregulated by the Mas receptor. Angiotensin II activates AT1a receptor signaling to drive pro-inflammatory pathways, promote abnormal ventricular remodeling and cardiac fibrosis, and trigger vasoconstriction, sympathetic activation, and hypertension (33, 34). In opposition, angiotensin-(1–7) activates vasoprotective Mas receptor signaling that exerts anti-inflammatory, anti-fibrotic, and anti-apoptotic actions in the heart (33, 34). Importantly, RAAS also regulates the pulmonary system. ACE, angiotensin II, and the AT1a receptor induce pro-inflammatory and fibrotic pathology in the lung, while ACE2, angiotensin-(1–7), and the Mas receptor protect the lung from inflammatory-induced injury and fibrosis during severe infection from pathogens including SARS-CoV-2 (35). Androgens testosterone and dihydrotestosterone have been reported to upregulate expression of angiotensinogen, renin, ACE, and angiotensin AT1 receptors (36, 37), which would produce a RAAS imbalance and may contribute to COVD-19 severity in men, an effect that would be ameliorated by ADT. Estradiol, however, upregulates expression of ACE-2, facilitates angiotensin-(1–7) formation and Mas receptor signaling, and downregulates angiotensin AT1a receptor expression, which may account for more favorable COVID-19 outcome in women (36–39).

An important caveat is that ADT may only be beneficial in the early phase of COVID-19 when T cell activation and other immune responses are critical (40, 41). Low testosterone levels during the late stages of COVID-19 infection have been associated with greater disease severity possibly due to the higher risk for cytokine storms without androgen immunosuppression (42). Genetics may also influence the effects of androgens on COVID-19 outcome. A trinucleotide (CAG) repeat sequence on exon 1 of the androgen receptor gene can modulate the function of the androgen receptor (AR) and serve as a genetic marker of androgen sensitivity. Shorter CAG repeat length increases AR sensitivity to testosterone and DHT, higher AR transcriptional activity, and a more active androgen receptor, while longer CAG repeat length reduces AR sensitivity and activity. Recently, androgen receptor CAG repeat length has been found to predict COVID-19 severity indicating the importance of genetically determined AR sensitivity in the mediation of androgen impact on COVID-19 outcome in men (43, 44). Other androgen genetics may also mediate COVID-19 infection and severity. Genome-wide association studies have identified genes strongly associated with testosterone levels. The VA Million Veteran Program MVP035 COVID-19 Disease Mechanisms study has constructed a testosterone polygenic risk score from GWAS data (45) and is now determining the role of testosterone gene variants in the COVID-19 severity. Androgen mechanisms contributing to COVID-19 will continue to require further investigation to elucidate their complexity.

## Data Availability Statement

The data analyzed in this study are subject to the following licenses/restrictions: Data restrictions apply as outlined in the Rules of Behavior for Access and Use of Data from VHA Vital Status File. The data are not publicly available. Further inquiries can be directed to the corresponding author.

## Ethics Statement

The studies involving human participants were reviewed and approved by the VA Central Institutional Review Board. Written informed consent for participation was not required for this study in accordance with the national legislation and the institutional requirements.

## Author Contributions

MR, KL, JAL, JL, M-CS, DB, SL, and DM contributed to conception and design of the study. KL, AG, and KH prepared the data and performed statistical analysis. MR wrote the first draft of the manuscript. KL, DB, SL, and RH wrote sections of the manuscript. All authors contributed to manuscript revision, read, and approved the submitted version.

## Author Disclaimer

The views expressed are those of the authors and do not necessarily represent the views or policy of the Department of Veterans Affairs or the United States Government.

## Conflict of Interest

SL reports equity in Gilead Sciences, Inc. SD reports research grants from the following for-profit organizations outside this submitted work: Alnylam Pharmaceuticals Inc., AbbVie Inc., Astellas Pharma Inc., AstraZeneca Pharmaceuticals LP, Biodesix, Inc., Boehringer Ingelheim International GmbH, Celgene Corporation, Eli Lilly and Company, Genentech Inc., Gilead Sciences Inc., GlaxoSmithKline PLC, Innocrin Pharmaceuticals Inc., Janssen Pharmaceuticals, Inc., Kantar Health, Myriad Genetic Laboratories, Inc., Novartis International AG, and Parexel International Corporation through the University of Utah or Western Institute for Veteran Research. The remaining authors declare that the research was conducted in the absence of any commercial or financial relationships that could be construed as a potential conflict of interest.

## Publisher’s Note

All claims expressed in this article are solely those of the authors and do not necessarily represent those of their affiliated organizations, or those of the publisher, the editors and the reviewers. Any product that may be evaluated in this article, or claim that may be made by its manufacturer, is not guaranteed or endorsed by the publisher.
