# Supplementary material for: A Population-Level Analysis of the Protective Effects of Androgen Deprivation Therapy Against COVID-19 Disease Incidence and Severity
Source: Front Med (Lausanne). 2022 May 4;9:774773. doi: 10.3389/fmed.2022.774773 (PMC9115469; doi:10.3389/fmed.2022.774773)
Supplement: Supplementary file 1 [file Data_Sheet_1.pdf]

SUPPLEMENTARY APPENDIX

Table S1. Baseline characteristics of base sample and matched sample
Table S2. Logistic regression results of propensity score model to predict receipt of SARS-CoV-2 testing
Table S3. Association between ADT use and COVID-19 positivity and severity among prostate cancer patients

Table S1. Baseline characteristics of base sample and matched sample

	Base				Matched			
	**Tested**		**Not tested**		**Case**		**Control**	
**Number of patients**	248,264		6,002,153		246,087		1,230,435	
**ADT (%)**	3,088	(1.2)	25,224	(0.4)	3,057	(1.2)	6,272	(0.5)
**Age (years) (%)**								
Mean (SD)	63.0	(15.0)	62.6	(16.8)	63.0	(15.0)	63.0	(15.0)
< 45	34,831	(14)	1,101,250	(18)	34,241	(14)	171,205	(14)
45-54	27,986	(11)	688,992	(11)	27,677	(11)	138,385	(11)
55-64	53,955	(22)	982,064	(16)	53,616	(22)	268,080	(22)
65-74	83,748	(34)	1,829,250	(30)	83,463	(34)	417,315	(34)
75-84	33,005	(13)	908,966	(15)	32,757	(13)	163,785	(13)
≥ 85	14,739	(6)	491,631	(8)	14,333	(6)	71,665	(6)
**Race (%)**								
White, non-Hispanic	152,651	(61)	4,016,395	(67)	152,601	(62)	763,005	(62)
White, Hispanic	15,260	(6)	284,011	(5)	14,848	(6)	74,240	(6)
Black	58,701	(24)	919,061	(15)	58,414	(24)	292,070	(24)
HI/PI	2,034	(1)	49,117	(1)	1,520	(1)	7,600	(1)
AI/AK	1,993	(1)	45,026	(1)	1,488	(1)	7,440	(1)
Asian	2,543	(1)	64,830	(1)	2,201	(1)	11,005	(1)

Unknown	15,082	(6)	623,713	(10)	15,015	(6)	75,075	(6)
**Marital status (%)**								
Married	113,540	(46)	3,397,095	(57)	112,531	(46)	669,013	(54)
Single	41,480	(17)	874,191	(15)	41,077	(17)	182,585	(15)
Separated or divorced	78,488	(32)	1,365,029	(23)	77,922	(32)	312,150	(25)
Widowed	12,927	(5)	251,705	(4)	12,751	(5)	48,705	(4)
Unknown	1,829	(1)	114,133	(2)	1,806	(1)	17,982	(1)
**BMI (%)**								
Mean (SD)	29.8	(6.4)	29.7	(5.8)	29.8	(6.4)	29.8	(5.9)
< 18.5	3,992	(2)	48,488	(1)	3,958	(2)	10,747	(1)
18.5-24.9	51,195	(21)	1,100,654	(18)	50,675	(21)	227,021	(18)
25.0-29.9	83,237	(34)	2,169,578	(36)	82,520	(34)	443,159	(36)
≥ 30	108,494	(44)	2,493,168	(42)	107,609	(44)	521,060	(42)
Unknown	1,346	(1)	190,265	(3)	1,325	(1)	28,448	(2)
**Smoking status (%)**								
Current	81,227	(33)	1,619,508	(27)	80,578	(33)	350,277	(28)
Former	132,108	(53)	2,970,410	(49)	130,969	(53)	608,266	(49)
Never	34,929	(14)	1,412,235	(24)	34,540	(14)	271,892	(22)
**CCI Categories (%)**								
Cerebrovascular disease	30,288	(12)	307,669	(5)	30,072	(12)	69,586	(6)
Congestive heart failure	39,713	(16)	307,897	(5)	39,421	(16)	71,017	(6)

Chronic pulmonary disease	67,640	(27)	758,051	(13)	67,200	(27)	166,445	(14)
Dementia	25,365	(10)	192,304	(3)	25,069	(10)	40,135	(3)
Diabetes without chronic complication	86,249	(35)	1,304,921	(22)	85,556	(35)	295,000	(24)
Diabetes with chronic complication	59,354	(24)	697,034	(12)	58,895	(24)	160,550	(13)
Hemiplegia or paraplegia	5,032	(2)	28,439	(0)	5,007	(2)	7,436	(1)
HIV/AIDS	3,048	(1)	22,628	(0)	3,029	(1)	6,966	(1)
Mild liver disease	30,345	(12)	269,707	(4)	30,120	(12)	69,213	(6)
Severe liver disease	15,095	(6)	110,360	(2)	14,996	(6)	31,710	(3)
Localized solid tumor	53,487	(22)	666,305	(11)	53,155	(22)	150,531	(12)
Metastatic solid tumor	6,115	(2)	30,613	(1)	6,078	(2)	7,609	(1)
Myocardial Infarction	15,474	(6)	104,337	(2)	15,376	(6)	25,004	(2)
Peptic ulcer disease	4,569	(2)	31,019	(1)	4,533	(2)	7,681	(1)
Peripheral vascular disease	37,885	(15)	363,926	(6)	37,622	(15)	81,429	(7)
Renal disease	55,037	(22)	541,912	(9)	54,600	(22)	122,707	(10)
Rheumatic disease	4,994	(2)	64,291	(1)	4,961	(2)	13,796	(1)
**Medications (%)**								
ACE	59,233	(24)	960,899	(16)	58,825	(24)	219,513	(18)
ARB	25,454	(10)	384,629	(6)	25,265	(10)	86,733	(7)
Spironolactone	8,447	(3)	78,357	(1)	8,403	(3)	18,440	(1)
**VHA utilization in the prior year**								
Outpatient visits (SD)	32.7	(36.2)	10.6	(15.5)	32.7	(36.2)	12.5	(17.8)

Inpatient days (SD)	6.9	(28.8)	0.7	(7.2)	6.9	(28.7)	1.0	(8.5)

Abbreviations: ADT, Androgen Deprivation Therapy; SD, Standard Deviation; BMI, Body Mass Index; CCI, Charlson Comorbidity Index; ACE, Angiotensin-converting enzyme inhibitors; ARB, Angiotensin II Receptor Blockers; VHA, Veterans Health Administration.

Table S2. Logistic regression results of propensity score model to predict receipt of SARS-CoV-2 testing

	Unadjusted	Fully adjusted	
	**OR (95% CI)**	**OR (95% CI)**	
**ADT**	2.46 (2.35,2.56)	1.59	(1.52,1.67)
**Age**		0.99	(0.99,0.99)
**Race**			
White, non-Hispanic (Ref).			
White, Hispanic		0.96	(0.94,0.98)
Black		0.79	(0.78,0.80)
Other		1.00	(0.97,1.03)
Unknown		1.27	(1.24,1.29)
**Marital status**			
Married (Ref.)			
Single		1.22	(1.20,1.24)
Separated or divorced		1.27	(1.26,1.28)
Widowed		1.34	(1.31,1.37)
Unknown		0.72	(0.68,0.76)
**BMI**		0.99	(0.99,1.00)
**Smoking status**			
Current (Ref.)			
Former		1.01	(1.00,1.02)
Never		0.82	(0.81,0.83)
**Comorbidities**			
Cerebrovascular disease		1.18	(1.16,1.20)
Congestive heart failure		1.30	(1.28,1.32)
Chronic pulmonary disease		1.45	(1.44,1.47)
Dementia		1.61	(1.57,1.64)

Diabetes without chronic complication	1.05	(1.04,1.07)
Diabetes with chronic complication	1.08	(1.06,1.09)
Hemiplegia or paraplegia	1.16	(1.11,1.21)
HIV/AIDS	1.40	(1.34,1.47)
Mild liver disease	1.35	(1.33,1.38)
Severe liver disease	0.95	(0.92,0.98)
Myocardial Infarction	1.22	(1.19,1.25)
Peptic ulcer disease	1.32	(1.27,1.38)
Peripheral vascular disease	1.23	(1.21,1.25)
Renal disease	1.30	(1.29,1.32)
Rheumatic disease	1.19	(1.15,1.23)
**Medications**		
ACE	1.14	(1.13,1.15)
ARB	1.13	(1.11,1.15)
Spironolactone	1.00	(0.97,1.03)
**VHA utilization in the prior year**		
Outpatient visit	1.03	(1.03,1.03)
Inpatient days	1.00	(1.00,1.00)

Abbreviations: ADT, Androgen Deprivation Therapy; SD, Standard Deviation; BMI, Body Mass Index; ACE, Angiotensin-converting enzyme inhibitors; ARB, Angiotensin II Receptor Blockers; VHA, Veterans Health Administration.

Table S3. Association between ADT use and SARS-CoV-2 positivity and COVID-19 severity among prostate cancer patients

	ADT		No ADT (reference)				
**Outcome**	**Event (%)**	**No. Tested/Positive**	**Event (%)**	**No. Tested/Positive**	**OR**	**(95% CI)**	**P-value**
SARS-CoV-2 positivity	149 (8)	1,908	1228 (10)	12,741	0.85	(0.77,0.94)	0.002
Severe COVID-19*	42 (22)	189	316 (23)	1,346	0.97	(0.65,1.45)	0.893

Abbreviations: ADT, Androgen Deprivation Therapy; SARS-CoV-2, Severe acute respiratory syndrome coronavirus 2; COVID-19, Coronavirus Disease 2019.

*Severe COVID-19 is defined as ICU admission, mechanical ventilation, or death in the 60 days following SARS-CoV-2 positivity.
